# Development of a Digital Health Literacy Assessment Framework for Older Adults: Delphi-Based Study

**DOI:** 10.2196/82334

**Published:** 2026-04-29

**Authors:** Xingxia Zhang, Yongqing Yuan, Jie Jiang

**Affiliations:** 1 West China Hospital, Sichuan University, No. 37 Guoxue Alley, Wuhou District, Chengdu, Sichuan, 610041, China, 86 18980602181

**Keywords:** older adults, digital health literacy, eHealth, evaluation, Delphi method, China

## Abstract

**Background:**

Digital health literacy (DHL) is crucial for improving health outcomes. As a digitally marginalized group, older adults face significant barriers in using digital technologies for health information access and eHealth services, which hinders their ability to benefit from digital dividends. A comprehensive and contextually relevant assessment tool is essential to understand their DHL and to inform effective interventions.

**Objective:**

This study aimed to develop a theoretically grounded and content-valid assessment framework for DHL among older adults in China, establishing a foundation for future measurement and research.

**Methods:**

A multiphase, theory-informed approach was adopted. The construct domains and an initial item pool were derived from a literature review and qualitative focus group discussions. A 2-round Delphi consultation was conducted to refine and reach consensus on the indicator system. Subsequently, a pilot cross-sectional survey was conducted to validate the reliability and validity of the scale.

**Results:**

Two rounds of expert consultation were conducted with 15 experts, and 322 older adult participants were included in the cross-sectional survey. The expert authority coefficients (*Cr*) were 0.748 and 0.768, respectively, and the Kendall harmony coefficient was statistically significant. The resulting “individual competency-environmental support” dual-pathway framework comprises 6 primary domains (confidence and trust in application, operational skills, critical evaluation skills, application skills, security and privacy, and external support), 13 subdomains, and 27 specific indicators. In the pilot sample (N=332), the overall framework showed excellent internal consistency, and the Cronbach α coefficients ranged from 0.913 to 0.987. Criterion validity was supported by a strong correlation with the established eHealth Literacy Scale tool (*r*=0.891; *P*<.001). Known-groups validity was confirmed, as the framework successfully discriminated between older adults with different educational levels (*t*_330_=−4.37; *P*<.001).

**Conclusions:**

This study developed a dual-dimensional “individual competency–environmental support” framework to assess DHL among older adults. The framework systematically identifies competency variations and contextual barriers, offering a standardized tool for needs assessment, intervention design, and evaluation of digital inclusion efforts. It also supports the development of targeted, multisectoral strategies to enhance digital health engagement in aging populations. Further validation across diverse settings is recommended to strengthen its generalizability and practical applicability.

## Introduction

With the rapid development of digital technology and the digital transformation of health care, digital access and skills are increasingly becoming fundamental social determinants of health, as the effective use of both social resources and health care information continues to migrate online in the digital era [1]. Digital health literacy (DHL), defined as the ability to search, filter, understand, evaluate, and apply health information obtained through electronic platforms to solve health-related issues [2], has evolved into a core competency for advancing health management and population health outcomes [3]. Studies have shown that DHL is positively correlated with health-related behaviors [4], self-health management [5], quality of life [6], and other health-related outcomes [7]. Digital literacy is a pivotal element in conditioning the effectiveness of telemedicine and digital medicine services, plays a critical role in achieving health equity in third-millennium society [8], and can reach large numbers of people at relatively low cost [9]. Nevertheless, ongoing digital divides hamper improvements in patient self-management, care coordination, quality, and cost-effectiveness [10], especially among marginalized populations, such as ethnic minority groups, individuals with limited English proficiency, those with low socioeconomic status, and older adults [11]. A scoping review revealed that research on DHL in older adults is still in its infancy, especially in terms of assessment tools and intervention methods [12]. As “digital refugees” (here, referring to individuals who, due to multidimensional capability deficits in accessing, comprehending, and applying digital health resources, experience systematic exclusion from digital health ecosystems and ultimately suffer technologically mediated health inequities through marginalization), the DHL needs of older adults warrant greater attention.

DHL quantitative assessment is a critical step in scientifically, objectively, and comprehensively evaluating the proficiency of specific populations, drawing significant attention from scholars in related fields. A recent bibliometric analysis of global eHealth highlighted the eHealth Literacy Scale (eHEALS) as both a pivotal instrument and a defining frontier issue for advancing research in this domain [13]. The measurement tools developed during different stages of internet evolution vary. In 2006, Norman and Skinner [14] labeled this ability “eHealth literacy” (EHL) and developed eHEALS, an 8-item measure of EHL developed to measure consumers’ combined knowledge, comfort, and perceived skills in finding, evaluating, and applying eHealth information to health problems, emphasizing the use of health information available on the internet. As the use of eHealth grows and diversifies globally, the eHEALS may not fully align with user-website interactions in Web 2.0 [15]. The core distinction between Web 2.0 and Web 1.0 lies in interactivity. Web 1.0 is characterized primarily by unidirectional dissemination, whereas Web 2.0 enables users to contribute user-generated content to the web, thereby establishing bidirectional engagement [16]. The measurement tools developed during Web 2.0 have broadened the multidimensional conceptualization of DHL through refined dimensional structuring, incorporating metrics assessing internet proficiency and online interactive communication capabilities [17]. Within the current Web 3.0 environment—the “semantic web” operating on a “read-write-execute” model to deliver digital, personalized, and intelligent services [18]—Liu et al [19] developed the 24-item 3D eHEALS-Web 3.0, including acquisition, verification, and application, which was subsequently validated among older adults with noncommunicable diseases [20]. Several measurement tools have been developed for the general population, such as the Digital Health Literacy Assessment [21], Joint Information System Committee [22], Digital Health Readiness Questionnaire [23], Digital Health Technology Literacy Assessment Questionnaire [24], Yongin Severance Digital Sensitivity Scale [25], and others.

However, only a few studies have assessed the use and appropriateness of these measurements for older populations [26]. The eHEALS is the most frequently used instrument for measuring digital literacy among older adults [26], and was developed 19 years ago to measure abilities related to page views [14]. As the definition of DHL is ever-evolving, it is necessary to reassess and enhance existing DHL measures for better scaling of current eHealth usage. Recently, Wang et al [27] developed a 39-item older DHL questionnaire structured around 6 dimensions (information, behavior, security, interaction, content, and attitudes) based on the digital competence framework (DigComp). Although a valuable contribution, its primary focus on individual competencies such as information, interaction, security, and so on, as well as its length, may limit feasibility for widespread geriatric assessment and ignore a critical determinant of digital engagement in later life: the enabling or constraining role of the external environment [27].

Social ecological theory posits that individual health behaviors and outcomes result from continuous interactions between personal attributes and multilevel environmental contexts, such as interpersonal networks, community resources, and policy environments [28]. Similarly, the digital health divide is a multilevel phenomenon spanning “access-usage-benefit,” influenced by factors ranging from intrapersonal skills to social, community, and environmental conditions [29]. The digital health divide is likely determined not only by the DHL of individuals but also by various other interacting factors, such as individual lifestyle factors, attitudes, social and community networks, and wider cultural and environmental conditions [30-32]. For older adults, DHL is not merely an individual skill set but is profoundly shaped by external support systems, such as the availability of community resources, reliable internet infrastructure, and facilitative social networks. Existing tools, including those specifically designed for older adults [33-35], focus on assessing individual skills but largely neglect this socioecological perspective, creating a measurement gap.

Given the limitations in validating existing tools for older adults, future research should evaluate the diagnostic accuracy of measurement tools in this population and investigate the impact of social determinants of health on such assessments to strengthen their clinical implementation [36]. Guided by the social ecological perspective, we reconceptualize DHL in older adults as a context-rooted, composite competence that integrates personal agency and environmental enablement, highlighting the interaction of “individual-environment.” Consequently, the core contribution of this research lies in constructing an innovative dual-pathway “individual competency-environmental support” assessment framework, formally establishing “environmental support” as a core, independently measurable dimension of equal importance to personal competency. Methodologically, through a rigorous Delphi expert consensus process, we developed a more concise 27-item measurement instrument, aiming to balance comprehensiveness with respondent-friendliness for older adults.

In summary, this study aims to develop a theoretically grounded, structurally clear, and pragmatically feasible assessment tool. This instrument can simultaneously reveal the strengths and weaknesses of older adults’ DHL at both the personal competency and environmental support levels. It is designed to provide more precise and comprehensive empirical evidence for formulating systematic public health strategies and community-based interventions in the Web 3.0 era, ultimately aimed at reducing the digital health divide and promoting health equity among the older adult population.

## Methods

### Study Design

**Figure 1.**
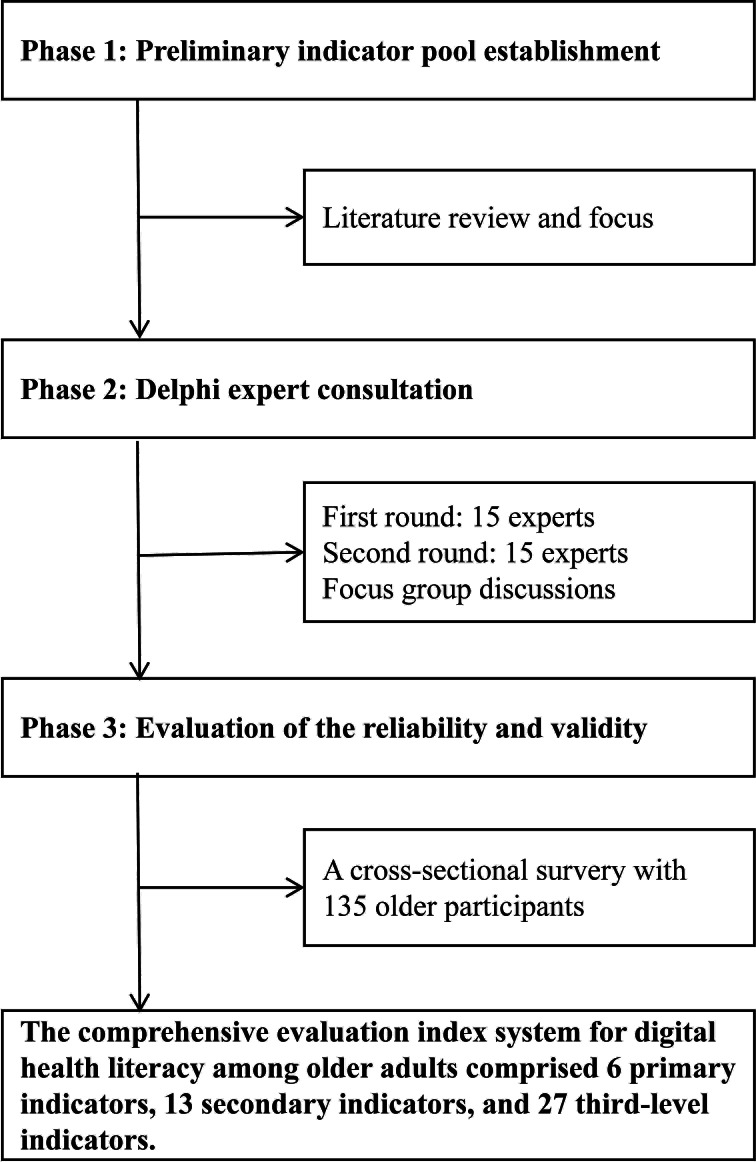
Research framework.

An initial evaluation index system was developed through a literature review and focus group discussions (FGDs). This was followed by a 2-round Delphi expert consultation involving 15 experts. The FGDs were convened by a 13-member research team led by the principal investigator, comprising 2 senior researchers, 4 midlevel researchers, 2 junior researchers, and 5 graduate researchers (2 doctoral and 3 master’s candidates). These discussions served 2 specific purposes: first, during the initial stage, they were used to screen and refine the item pool generated from the literature review, where participants discussed the relevance and clarity of potential items; second, during the Delphi process, they were used to facilitate discussion and decision-making regarding the adoption of expert opinions, helping to interpret disagreements and reach consensus on item modifications. Thus, the FGD data were iteratively used to inform item refinement and integrate expert feedback, rather than being analyzed through formal qualitative methods such as thematic or content analysis. By integrating qualitative analysis with quantitative computation, the initial framework comprising “6 primary indicators, 13 secondary indicators, and 28 tertiary indicators” was progressively refined into a scientifically structured system featuring “6-13-27” indicators. Finally, the reliability and validity of the questionnaire were verified through a cross-sectional survey. The specific procedural design is illustrated in Figure 1.

### Development of the Preliminary Indicator Pool

A comprehensive literature search was performed in the PubMed, Web of Science, Cochrane Library, CNKI, and WanFang databases to collect initial evaluation indicators using the following keywords:

“older adults” OR “older people” OR “elderly” AND “digital health literacy” OR “electronic health literacy” OR “eHealth literacy” OR “digital health” OR “information literacy” AND “indicator” OR “index” OR “scale.”

The search covered literature published from its inception to February 28, 2025. The results revealed that previous studies focused primarily on assessing the ability to access, evaluate, and apply health information, and ignored the influence of self-efficiency, as well as social and environmental elements, on DHL among older adults. Based on these findings, we developed a new comprehensive questionnaire with particular emphasis on the above dimensions, considering both the individual capacity and the environmental support of the older population.

### Using the Delphi Method to Develop an Index System for Expert Selection

This study used the Delphi method to administer expert consultation questionnaires via an online platform [37] to gather perspectives among participants, thereby enhancing the objectivity and scientific validity of the outcomes. While the Delphi methodology imposes no fixed threshold for expert panel size, increased participant numbers strengthen result stability [38]. A thorough review of the literature indicates that a panel of 4 to 16 experts generally yields sufficiently reliable findings for Delphi consultations. When the number of selected experts approaches 15, further increases contribute minimally to prediction precision. Consequently, this study ultimately enrolled 15 experts as participants for the consultation.

eHealth literacy represents a complex, multidisciplinary issue. The experts selected for consultation should possess authoritativeness, representation, and reliability, particularly to demonstrate substantial knowledge or experience relevant to the research topic. Based on the study’s objectives and content, the Delphi expert panel was recruited through purposive sampling, leveraging the research team’s established professional networks in geriatric care, clinical practice, and academic research. Invitations were extended to individuals who met the following predefined eligibility criteria: (1) work experience of 10 years or more in fields including geriatric medicine, geriatric nursing, older adult care services, or community management; (2) a bachelor’s degree or higher; (3) an intermediate-level technical title or higher; and (4) voluntary participation in the study with the ability to complete and return questionnaires promptly. Ultimately, a multidisciplinary panel of 15 experts including clinicians, nurses, health care administrators, and researchers, agreed to participate and completed both rounds of the Delphi consultation.

### Design of the Expert Consultation Questionnaire

Based on the findings from the literature review and qualitative interviews, the initial evaluation index system for assessing older adults’ EHL was developed into a consultation questionnaire. This instrument comprises four main sections: (1) an introduction to the research, including the purpose of the study, instructions for completing the questionnaire, details on how and when to submit it, contact information, and acknowledgments; (2) demographic information of experts; (3) the core section (expert consultation questionnaire on DHL evaluation indicators for urban community-dwelling older adults); and (4) an expert authority questionnaire, which includes expert self-assessments of their authority and familiarity with the questionnaire content according to theoretical analysis, work experience, peer understanding, and intuition.

Within the questionnaire’s primary section, the experts rated the importance of each indicator across all hierarchical levels via a 5-point Likert scale (ranging from 1=“Not Important” to 5=“Extremely Important”). Additionally, experts were invited to provide specific suggestions for adding, deleting, or modifying indicators in designated comment fields.

Two rounds of consultation were conducted via email and WeChat. A mean importance score of more than 3.50, a coefficient of variation (CV) of 0.30 or less, and a total score rate of more than 20% were the inclusion criteria [39,40]. Entries were adjusted on the basis of discussions within the research team, incorporating expert modifications. After 2 rounds, the final DHL questionnaire for older adults included 6 dimensions and 27 items (Table 1). The detailed results of scoring of experts were presented in the Multimedia Appendix 1.

**Table 1.** The comprehensive evaluation index system for digital health literacy (DHL) in older adults.

Tier-3 indicator	Importance, mean (SD)	CV^a^	Full score (%)
Confidence and trust in application	4.267 (0.884)	0.21	53.30
1.1 Belief in self-capability	4.333 (0.724)	0.17	46.70
1.1.1 I believe my actions can improve health status	4.400 (0.632)	0.14	46.70
1.2 Belief in online health information	4.200 (0.775)	0.18	40
1.2.1 I believe I can effectively obtain health-related information or services via the internet	4.267 (0.594)	0.14	33.30
1.2.2 I believe I can resolve health concerns using online resources	4.133 (0.743)	0.18	33.30
1.2.3 I am confident in applying online information for health decisions	4.000 (0.845)	0.21	33.30
Operational skills	4.533 (0.640)	0.14	60
2.1 Electronic device proficiency	4.333 (0.724)	0.17	46.70
2.1.1 I can input text or voice via mobile or computer keyboards	4.200 (0.862)	0.21	46.70
2.1.2 I can locate and follow health information platforms (eg, WeChat public accounts)	4.200 (0.862)	0.21	46.70
2.2 Health communication	4.467 (0.743)	0.17	60
2.2.1 I can clearly articulate health concerns in writing	4.333 (0.724)	0.17	46.70
2.2.2 I can verbally express symptoms and desired health solutions	4.467 (0.640)	0.14	53.30
2.3 Information retrieval	4.333 (0.724)	0.17	46.70
2.3.1 I use multiple online channels for diverse health information needs	4.533 (0.743)	0.16	66.70
2.3.2 I use accurate keywords for targeted information searches	4.400 (0.632)	0.14	46.70
Critical evaluation skills	4.333 (0.724)	0.17	46.70
3.1 Relevance assessment	4.400 (0.828)	0.19	60
3.1.1 I evaluate information applicability to my health condition	4.467 (0.640)	0.14	53.30
3.1.2 I identify appropriate health behaviors from retrieved information	4.333 (0.617)	0.14	40.00
3.2 Reliability assessment	4.533 (0.640)	0.14	60
3.2.1 I verify information authenticity	4.333 (0.724)	0.17	46.70
3.2.2 I discern commercial motives (eg, advertising) in information	4.200 (0.862)	0.21	46.70
3.3 Authenticity verification	4.667 (0.617)	0.13	73.30
3.3.1 I cross-verify information across websites	4.400 (0.828)	0.19	60
3.3.2 I discuss online health information with family, friends, or medical professionals for credibility assessment	4.467 (0.743)	0.17	60
Application skills	4.533 (0.640)	0.14	60
4.1 Practical use	4.333 (0.900)	0.21	53.30
4.1.1 I apply retrieved health information in daily life	4.333 (0.816)	0.19	53.30
4.1.2 I use information for health decisions (nutrition, medication, or emergency response)	4.200 (0.775)	0.18	40
Security and privacy	4.333 (0.976)	0.23	60
5.1 Protective awareness	4.400 (0.828)	0.19	60
5.1.1 I identify unsafe websites or social platforms	4.533 (0.915)	0.20	73.30
5.1.2 I protect personal/others’ private information (name or address)	4.667 (0.724)	0.16	80
5.1.3 I regularly clear browsing history	3.533 (1.060)	0.30	20.00
External support	4.000 (1.000)	0.25	40
6.1 Providers (community or health care institutions)	3.933 (0.799)	0.20	26.70
6.1.1 I access local health care services for health needs	4.600 (0.632)	0.14	66.70
6.1.2 I use community-based technology training for skill enhancement	4.270 (0.800)	0.19	46.70
6.2 Institutional support (government or enterprises)	4.000 (1.000)	0.25	40
6.2.1 My residence or senior centers have broadband internet	3.800 (1.080)	0.29	40
6.2.2 My devices have senior-friendly features (large text, icons, audio, or remote assistance)	3.930 (0.800)	0.20	26.70
6.3 Social support (family or community organizations)	4.130 (0.830)	0.20	40
6.3.1 I regularly interact with family online (eg, WeChat) or offline	4.270 (0.800)	0.19	46.70
6.3.2 I regularly interact with friends/community volunteers online or offline	4.130 (0.830)	0.20	40

a CV: coefficient of variation.

### Evaluation of the Reliability and Validity of the Index System

Participants and Sample Selection Permanent residents aged 60 years and older in a city in Sichuan Province (defined as people living in the city for 6 months or more, without distinguishing local household registration); basic understanding ability, ability to understand and fill in or answer the questions in the questionnaire; and voluntary participation in this study.

#### Sample Size

The 27 evaluation indicators were translated into specific questions to develop an empirical survey questionnaire. All response options used a 5-point Likert scale, where participants selected the score corresponding to their situation. Regarding the pilot survey sample size, a common rule is 5 to 10 participants per item [41]. An additional 20% buffer was included to account for potentially invalid responses. The target sample size was calculated as: 27 items*10 participants/item*(1+20%)=324 participants. The pilot study successfully recruited 332 community-dwelling older adults from Pengzhou city, Sichuan Province, meeting and slightly exceeding this target.

#### Survey Tools

The questionnaire encompassed four sections: (1) questionnaire instructions and informed consent, (2) general information about the respondents (designed by the researcher, including age, sex, place of residence, education level, marital status, household size, living arrangement, and so on), (3) the eHealth Literacy Assessment Scale, and (4) the culturally adapted Chinese version of the eHEALS [42] (developed by Norman et al [14]), which is an 8-item measure of eHealth literacy developed to measure consumers’ combined knowledge, comfort, and perceived skills in finding, evaluating, and applying eHealth information to health problems, with scores ranging from 1 to 5 points according to the Likert 5-level scoring method, and the total score is 8 to 40 points (Multimedia Appendix 2).

### Ethical Considerations

The study was approved by the ethics committee on Biomedical Research, West China Hospital of Sichuan University (1044). All participants were informed of the study’s purpose, and procedures, and provided voluntary consent to participate. Delphi experts received an honorarium for their participation, while cross-sectional survey participants took part voluntarily and received no compensation. The survey adhered to the principles of anonymity and confidentiality, and all responses were collected anonymously.

### Reliability and Validity Testing

The Cronbach α coefficient and Spearman-Brown split-half reliability are the most commonly used methods for testing the internal consistency reliability of questionnaires [43]. The split-half reliability was assessed by dividing the 27 items into 2 halves using the odd-even method. Generally, a Cronbach α coefficient greater than 0.500 for each dimension, along with a total Cronbach α coefficient and Spearman-Brown split-half reliability greater than 0.7 indicate acceptable reliability.

For validity testing, since there is currently no universally established “gold standard” instrument specifically designed to measure comprehensive DHL within the contemporary Web 3.0 paradigm for older adults, the eHEALS was used as the criterion tool in this study. Criterion validity was assessed by examining the correlation between the total score of our scale and the eHEALS score. In addition, known-groups validity was evaluated. Participants were divided into 2 groups based on their educational attainment (high school or above vs junior high school or below), as educational level has been consistently identified as a key determinant of DHL in previous literature [44]. An independent-samples 2-tailed *t* test was conducted to compare the mean scale scores between the 2 groups. Construct validity, assessed through factor analysis, was not performed in this phase of the study, primarily due to the limited sample size. This limitation is acknowledged in the “Discussion” section and will be addressed in future research with a larger sample.

### Statistical Analysis

Data management and statistical analyses were conducted using Microsoft Excel and the SPSSAU online platform (Beijing Qingsi Technology Co, Ltd). Expert demographic characteristics were described with frequencies (percentages) for categorical variables and means (SDs) for continuous variables. Expert engagement was evaluated through questionnaire response rates and the proportion of experts providing substantive feedback. Expert authority was quantified via the authority coefficient (Cr=[Ca+Cs]/2). The judgment basis coefficient (Ca) was derived from the expert’s self-assessment of the influence level (high, medium, or low) across 4 dimensions, with scores assigned as shown in Table 2.

**Table 2.** Assignment of values for the familiarity coefficient (Cs).

Basis of judgment	Major value	Medium value	Minor value
Practical experience	0.3	0.2	0.1
Theoretical analysis	0.5	0.4	0.3
Literature review	0.1	0.1	0.1
Intuitive judgment	0.1	0.1	0.1

The familiarity coefficient (Cs) was assigned based on the expert’s self-rated familiarity with the subject, using a 5-point scale (1.0=“very familiar,” 0.75=“familiar,” 0.5=“generally familiar,” 0.25=“somewhat unfamiliar,” and 0=“very unfamiliar”). Both components were collected via a self-assessment questionnaire completed by the experts. Consensus was assessed via the CV and the Kendall concordance coefficient (*w*).

## Results

### Results of the Delphi Consultation

The Delphi consultation involved 15 experts from 13 universities and tertiary-level hospitals located in Sichuan Province, Beijing, Shanghai, Hunan Province, Shandong Province, and Xi’an Province, China. All 15 (100%) completed both rounds of the Delphi consultation. Among the 15 experts, 6 (40%) were male participants and 9 (60%) were female participants. The participants were aged 36 to 56 years (mean 45.93, SD 6.17), with professional experience ranging from 10 to 34 years (mean 21.07, SD 8.21). Twelve (80%) experts held a master’s degree or higher, and 13 (86.67%) possessed associate senior professional titles or above. Regarding administrative roles, 1 (6.67%) served as a hospital president, 8 (53.33%) held positions as directors or deputy directors of clinical departments, and 1 (6.67%) was the head nurse of a ward unit. The characteristics are presented in Table 3.

**Table 3.** Characteristics of the Delphi participants (N=15).

Characteristics	Value, n (%)
Sex	
Male	6 (40)
Female	9 (60)
Age (y)	
≤40	2 (13.33)
41‐49	9 (60)
50‐60	4 (26.67)
Education	
Undergraduate	3 (20)
Master’s	2 (13.33)
PhD	10 (66.67)
Profession title	
Middle	2 (13.33)
Vice senior	8 (53.33)
Full senior	5 (33.33)
Administrative positions	
Hospital-level leadership	1 (6.67)
Director of department	3 (20)
Deputy director	5 (33.33)
Head nurse	1 (6.67)
None	5 (33.33)
Years worked	
10‐20	2 (13.33)
21‐29	10 (66.67)
≥30	3 (20)
Ares of expertise	
Health care services	8 (53.33)
Integrated older adult care	3 (20)
Public health	2 (13.33)
Geriatrics	1 (6.67)
Medical informatics	1 (6.67)

### Expert Engagement

A questionnaire return rate exceeding 70% is generally considered indicative of good retrieval and high expert engagement [45]. In the first round of this study’s expert consultation, 15 expert correspondence questionnaires were distributed, and all 15 were returned, yielding a return rate of 100%. Among these, 7 (46.67%) experts provided comments. In the second round, 15 questionnaires were distributed, all 15 were returned (100% return rate), and 4 (26.67%) experts provided comments. These results demonstrate a high level of expert engagement in the consultation process.

### Expert Authority

The expert authority coefficient (Cr) is determined jointly by Cs and Ca. The mean Cr values for the 2 consultation rounds were 0.748 (SD 0.096) and 0.768 (SD 0.089), both exceeding the acceptable threshold of 0.7 [45]. Therefore, the expert authority level for this consultation is deemed acceptable.

### Expert Consensus

Following 2 rounds of consultation, expert opinions converged, demonstrating high acceptance of all indicators. The CV in the first round ranged from 0.141 to 0.207, whereas in the second round it ranged from 0.141 to 0.225. The Kendall *W* coefficient tests for both consultation rounds yielded statistically significant results (*P*<.001 and *P*=.005, respectively), indicating strong consensus among expert evaluations.

### Modification of Indicators and the Evaluation Index System

In the first Delphi round, the definition of 1 primary indicator was revised, and the conceptual definition of “confidence and trust in application” was redefined as “trust in self-efficacy and online health information.” One secondary indicator (“belief in online health” was updated to “belief in online health information”) underwent revisions, while 1 tertiary item was eliminated because of content overlap with the 12 tertiary items that were modified.

In the second round, 1 primary indicator definition was refined, and the definition of the primary dimension “external support” was refined from “evaluation of external factors” to “perception of external factors.” One secondary indicator was amended, and minor wording optimizations were implemented for 6 tertiary items.

All indicators achieved a mean importance score exceeding 3.5, with a full-score rate surpassing 20%, meeting predetermined criteria except for a few items demonstrating CV values below 0.30. Given the critical significance of privacy exposure and digital technology infrastructure in the digital era for DHL, the item (“5.1.3 I regularly clear browsing history”) was retained following rigorous FGDs. A strong perspective held that, in the context of managing online health information, this behavior concretely reflects privacy protection awareness—a critical facet of the “safety” dimension in DHL. Its high importance score and theoretical alignment supported its inclusion.

Through research team deliberation guided by predefined inclusion criteria and expert feedback, the final hierarchical framework for assessing older adults’ DHL comprised 6 primary indicators, 13 secondary indicators, and 27 third-level indicators (Table 1).

### Results of the Reliability and Validity of the Questionnaire

The pretest yielded 332 valid questionnaires. The participants’ ages ranged from 60 to 82 years, with a mean age of 65.4 (SD 5.2) years. The cohort comprised 175 (52.7%) males and 157 (47.3%) females. Married individuals constituted the majority (n=276, 83.1%). The education level was predominantly junior high school or below (n=243, 73.2%), and farming was the primary occupation (n=165, 49.7%). Most households consisted of 3 to 5 members (n=233, 70.1%), and coresiding with children was the most common living arrangement (n=176, 53%). The detailed characteristics are presented in Table 4.

**Table 4.** Characteristics of the participants (N=332).

Characteristics		Values
Age (y), mean (SD)		65.41 (5.18)
Sex, n (%)		
Male		175 (52.7)
Female		157 (47.3)
Marital status, n (%)		
Married		276 (83.1)
Widowed		41 (12.3)
Divorced		12 (3.6)
Unmarried		3 (0.9)
Education level, n (%)		
Junior high school or below		243 (73.2)
High school		73 (22.0)
College		16 (4.8)
Occupation, n (%)		
Farmer		165 (49.7)
Retired		124 (37.3)
Self-employed		38 (11.4)
Other		5 (1.5)
Health insurance, n (%)		
Urban-rural resident basic medical insurance		209 (63.0)
Employee basic medical insurance		123 (37.0)
Household size (persons), n (%)		
<3		53 (16.0)
3‐5		233 (70.1)
>5		46 (13.9)
Living arrangement, n (%)		
Co-residing with children		176 (53.0)
Co-residing with partner		128 (38.6)
Living alone		24 (7.2)
Other		4 (1.2)

### Scoring, Weighting, and Preliminary Interpretation of the Index System

The instrument is based on a 5-point Likert scale. The total score is derived from the sum of all 27 items, with a theoretical range of 27 to 135 points. In the validation phase, an equally weighted summed score was used for clarity and ease of interpretation, with each item contributing equally to the total.

In the cross-sectional survey, the mean total DHL score among older adults was 70.13 (SD 23.53), with a median score of 67.0 (IQR 51.0‐91.0). The mean item score was 2.60 (SD 0.87), with a median item score of 2.48 (IQR 1.89‐3.37), based on a 5-point Likert scale.

### Result of the Reliability Analysis

This study primarily assessed the internal consistency reliability and split-half reliability of our DHL for older adults. The IBM SPSS software yielded the following results: internal consistency reliability: The overall Cronbach α coefficient for DHL was 0.984, exceeding the acceptable threshold of 0.70. All dimensions demonstrated satisfactory internal consistency, with Cronbach α coefficients exceeding the threshold of 0.70. The detailed results are presented in Table 5.

**Table 5.** Results of the reliability analysis.

Dimension	Cronbach α	Number of items
Confidence and trust in application	0.968	4
Operational skills	0.966	6
Critical evaluation skills	0.987	6
Application skills	0.913	2
Security and privacy	0.935	3
External support	0.954	6
Split-half reliability		
First half	0.968	14
Second half	0.967	13
Full scale	0.984	27

### Split-Half Reliability

The split-half reliability was assessed by dividing the 27 items into 2 halves using the odd-even method. The Cronbach α coefficients for these 2 subsets were 0.968 and 0.967, respectively. The correlation coefficient was 0.952. The Spearman-Brown split-half reliability coefficient for the full scale was 0.922. These results collectively demonstrate excellent internal consistency reliability for DHL.

### Results of the Validity Analysis

Criterion validity was used to evaluate the validity of the DHL. The Chinese version of the eHEALS serves as the reference criterion, as it represents one of the most widely used and validated instruments in this domain [26]. Its established reliability in older Chinese populations (Cronbach *α*=0.988) [46] supports its suitability as a benchmark. Analysis revealed a strong Pearson correlation coefficient of 0.891 (*P*<.001) between the DHL scale and the eHEALS, demonstrating good concurrent validity for the newly developed scale.

To further examine the construct validity of the scale, a known-groups validity analysis was conducted. Consistent with existing literature [44], educational attainment is a well-established predictor of DHL. Participants were therefore divided into 2 groups based on their level of education: “high school or above” education (n=89) and a group with “junior high school or below” education (n=243). The results of an independent samples *t* test revealed a statistically significant difference between the groups (*t*_330_=−4.37; *P*<.001). This finding indicates that the newly developed scale can effectively discriminate between groups with theoretically different levels of DHL, thereby providing strong evidence for its construct validity.

## Discussion

### Principal Findings

In this study, an assessment framework and corresponding scale for evaluating the DHL of older adults were developed and underwent preliminary validation. The framework comprises 2 dimensions—individual competency and external support—with 6 first-level indicators, 13 second-level indicators, and 27 third-level indicators, covering the core dimensions of DHL among older adults. The total score ranges from 27 to 135, derived from the sum of all 27 items rated on a 5-point Likert scale. To offer an initial reference for application, an interpretive framework was developed based on the percentile distribution of the pilot sample (N=332). Specifically, total scores below the 25th percentile were preliminarily categorized as “needing support,” scores between the 25th and 75th percentiles as “having foundational competency,” and scores above the 75th percentile as “proficient.” It must be emphasized that this framework and its cutoff values are derived from the pilot data and serve as provisional, practical benchmarks. Their generalizability and optimal thresholds require further validation in future studies with larger, more representative populations.

The study fully considers the interplay between socioecological levels, and the finalized “individual competency-environmental support” dual-pathway index system transcends the limitations of conventional measurement tools that focus solely on individual skills. At the individual competency level, the newly incorporated “confidence and trust in application” dimension emphasizes self-efficacy and trust in online health information, capturing psychological and cognitive influences on digital health behaviors. The “critical evaluation skills,” “application skills,” and “security and privacy” dimensions delineate competencies in information filtering, authenticity verification, and privacy protection, aligning with digital health demands in the Web 3.0 era. At social and community levels, the influence of family, friends, and peers on an individual’s health-related behavior has been recognized [47,48]. In the DHL scale, the wider cultural, social, and environmental level elements are included. The external support dimension encompasses multiple stakeholders (community, government, institutions, family, and so on), where indicators such as “health care services,” “community-based technology training,” and “broadband internet” specifically address practical barriers to digital inclusion, providing a clear direction for policy development and service design.

### Comparison With Prior Work

There are many measurements in the field of DHL. However, only a few studies have explored the influencing factors of older adults’ DHL from the perspective of social capital [49]. Compared with existing instruments, the key distinction of this framework lies in its systematic incorporation of “environmental support” as an independent dimension, moving beyond a focus solely on individual skills. While widely used tools such as eHEALS emphasize personal operational and cognitive abilities, this study integrates external factors, including community, family, and social support, aligning more closely with health ecological theory and digital inclusion perspectives. Methodologically, the use of multiround Delphi consensus combined with qualitative data from focus groups enhanced the content validity and cultural relevance of the indicators, addressing limitations associated with purely literature-driven or statistically screened item pools, which are commonly used in the research of tool construction [25,50].

This assessment framework has dual practical significance. First, it establishes a standardized screening tool for assessing older adults’ DHL. The external support was first included in the DHL evaluation index system. Second, it promotes multistakeholder collaboration to enhance digital health capabilities. In addition to increasing eHealth skills, it is important to consider the role of trust, self-efficacy, motivation, social influence, and support in the use of digital health tools [51]. In terms of government, policies on national digital infrastructure (eg, free Wi-Fi for all), health service budgets, and resources for investing in and supporting digital health rollout can be used to mitigate digital inequalities [11], and communities and families can implement targeted training and companion services [52]. This integrated approach empowers older adults to transition from “digitally vulnerable groups” to “active health self-managers,” advancing the achievement of healthy aging goals.

### Limitations

Despite yielding noteworthy findings, this study has several limitations. First, methodologically, the scale relies on self-report and lacks item weighting, which may affect the objectivity of the results and the discriminant validity among core competencies. Future work will combine behavioral observation with confirmatory factor analysis to cross-validate the measurement approach and explore weighting schemes. Second, the single-center, cross-sectional design limits the generalizability of the findings and prevents examination of the tool’s ability to predict health behaviors. A multicenter longitudinal study is planned to expand sample diversity and track the relationship between scale scores and actual health behaviors. Third, the current framework lacks the capacity for dynamic assessment. Given the rapid advancement of digital technologies and the evolving health needs of older adults, the existing indicators may not remain timely or fully applicable in the long term [53]. A standing revision panel comprising experts in geriatrics, digital technology, and public health will be established to implement periodic evaluation and content updates, ensuring the tool’s continued relevance amid technological change.

### Conclusions

The dual-dimensional “individual competency–environmental support” framework developed in this study offers a structured tool for systematically identifying variations in digital health competencies among older adults. It highlights the combined role of individual and environmental factors in shaping DHL and provides a standardized approach to assess key competencies and contextual barriers. The framework can be used for needs identification, community-based intervention planning, and evaluation of digital inclusion initiatives, while also supporting the design of targeted, multistakeholder interventions. Importantly, this study aligns closely with national strategies such as “Healthy China” and “Active Aging,” offering evidence-based support for policy implementation from the perspective of digital empowerment and inclusive development. By promoting DHL among older adults, the research contributes to enhancing their ability to manage health autonomously and participate socially, thereby supporting the policy goal of building an aging-friendly digital health environment and an inclusive aging society. Future studies should validate the framework across diverse populations and settings to strengthen its applicability and policy relevance, ensuring its continued use in supporting and refining national healthy aging strategies.

## Supplementary material

10.2196/82334 Multimedia Appendix 1 Delphi expert rating scores for all questionnaire items.

10.2196/82334 Multimedia Appendix 2 Survey questionnaire on digital health literacy among urban and rural residents in Sichuan Province.

## Abbreviations

CV: coefficient of variation
DHL: digital health literacy
DigComp: digital competence framework
eHEALS: eHealth Literacy Scale
EHL: eHealth literacy
FGD: focus group discussion
