# Supplementary material for: Development of a Digital Health Literacy Assessment Framework for Older Adults: Delphi-Based Study
Source: J Med Internet Res. 2026 Apr 29;28:e82334. doi: 10.2196/82334 (PMC13127856; doi:10.2196/82334)
Supplement: Multimedia Appendix 1 [file jmir-v28-e82334-s001.doc]

Supplement materials. Delphi expert rating scores for all questionnaire items

| Experts/  Items | 1 | 2 | 3 | 4 | 5 | 6 | 7 | 8 | 9 | 10 | 11 | 12 | 13 | 14 | 15 | 16 | 17 | 18 | 19 | 20 | 21 | 22 | 23 | 24 | 25 | 26 | 27 | 28 | 29 | 30 | 31 | 32 | 33 | 34 | 35 | 36 | 37 | 38 | 39 | 40 | 41 | 42 | 43 | 44 | 45 | 46 |
| --- | --- | --- | --- | --- | --- | --- | --- | --- | --- | --- | --- | --- | --- | --- | --- | --- | --- | --- | --- | --- | --- | --- | --- | --- | --- | --- | --- | --- | --- | --- | --- | --- | --- | --- | --- | --- | --- | --- | --- | --- | --- | --- | --- | --- | --- | --- |
| A | 5 | 5 | 5 | 5 | 5 | 5 | 5 | 5 | 5 | 5 | 4 | 5 | 5 | 5 | 5 | 5 | 5 | 5 | 5 | 5 | 5 | 5 | 5 | 5 | 5 | 4 | 5 | 5 | 5 | 5 | 5 | 5 | 5 | 5 | 5 | 5 | 5 | 5 | 5 | 2 | 5 | 5 | 5 | 3 | 5 | 5 |
| B | 5 | 5 | 5 | 5 | 5 | 5 | 5 | 4 | 5 | 5 | 5 | 5 | 5 | 5 | 5 | 5 | 5 | 5 | 5 | 4 | 4 | 4 | 4 | 4 | 5 | 5 | 5 | 5 | 5 | 5 | 5 | 5 | 5 | 5 | 5 | 5 | 4 | 5 | 5 | 5 | 5 | 5 | 5 | 5 | 5 | 5 |
| C | 4 | 5 | 4 | 4 | 4 | 4 | 3 | 4 | 4 | 5 | 4 | 5 | 4 | 4 | 5 | 5 | 3 | 4 | 4 | 4 | 4 | 5 | 4 | 4 | 4 | 4 | 5 | 4 | 4 | 5 | 4 | 4 | 3 | 4 | 5 | 4 | 4 | 5 | 5 | 4 | 3 | 3 | 3 | 4 | 5 | 4 |
| D | 5 | 5 | 4 | 5 | 3 | 3 | 4 | 4 | 5 | 5 | 4 | 5 | 5 | 5 | 4 | 3 | 3 | 3 | 3 | 4 | 5 | 4 | 4 | 5 | 5 | 5 | 4 | 5 | 5 | 5 | 4 | 4 | 3 | 3 | 4 | 5 | 4 | 4 | 3 | 3 | 4 | 4 | 3 | 3 | 3 | 3 |
| E | 5 | 4 | 3 | 4 | 2 | 2 | 5 | 5 | 4 | 4 | 4 | 3 | 4 | 5 | 2 | 3 | 3 | 2 | 4 | 5 | 4 | 4 | 3 | 5 | 3 | 4 | 4 | 5 | 4 | 4 | 4 | 5 | 3 | 4 | 4 | 4 | 3 | 2 | 5 | 3 | 4 | 4 | 2 | 4 | 4 | 4 |
| F | 3 | 4 | 5 | 4 | 3 | 4 | 4 | 3 | 4 | 4 | 4 | 5 | 5 | 5 | 4 | 3 | 4 | 4 | 4 | 4 | 3 | 3 | 3 | 4 | 4 | 5 | 5 | 4 | 4 | 5 | 5 | 5 | 5 | 5 | 5 | 4 | 4 | 3 | 3 | 2 | 4 | 4 | 4 | 4 | 4 | 4 |
| G | 3 | 4 | 4 | 4 | 4 | 4 | 4 | 4 | 3 | 3 | 3 | 4 | 4 | 4 | 4 | 4 | 4 | 3 | 4 | 4 | 4 | 3 | 3 | 3 | 3 | 3 | 3 | 3 | 4 | 4 | 4 | 4 | 4 | 3 | 4 | 4 | 4 | 5 | 5 | 4 | 5 | 4 | 3 | 3 | 3 | 3 |
| H | 5 | 5 | 5 | 5 | 5 | 5 | 5 | 5 | 5 | 5 | 5 | 5 | 5 | 5 | 5 | 5 | 5 | 5 | 5 | 5 | 5 | 5 | 5 | 5 | 5 | 5 | 5 | 5 | 5 | 5 | 5 | 5 | 5 | 5 | 5 | 5 | 5 | 5 | 5 | 5 | 5 | 5 | 5 | 5 | 5 | 5 |
| I | 5 | 5 | 5 | 5 | 5 | 5 | 5 | 5 | 5 | 5 | 5 | 5 | 5 | 5 | 5 | 5 | 4 | 5 | 5 | 5 | 4 | 4 | 5 | 5 | 4 | 4 | 4 | 5 | 4 | 4 | 4 | 4 | 5 | 4 | 4 | 5 | 4 | 5 | 5 | 4 | 5 | 5 | 3 | 4 | 5 | 5 |
| J | 4 | 5 | 5 | 5 | 4 | 4 | 4 | 4 | 5 | 5 | 5 | 4 | 4 | 5 | 5 | 4 | 4 | 4 | 5 | 4 | 4 | 4 | 4 | 5 | 5 | 5 | 5 | 5 | 5 | 4 | 4 | 4 | 4 | 5 | 5 | 5 | 5 | 4 | 4 | 4 | 5 | 4 | 3 | 4 | 5 | 5 |
| K | 5 | 5 | 5 | 5 | 5 | 5 | 5 | 5 | 5 | 5 | 5 | 5 | 5 | 5 | 5 | 5 | 5 | 5 | 5 | 5 | 5 | 5 | 5 | 5 | 5 | 5 | 5 | 5 | 4 | 5 | 5 | 5 | 5 | 5 | 5 | 5 | 5 | 5 | 5 | 4 | 4 | 5 | 5 | 5 | 5 | 5 |
| L | 5 | 4 | 4 | 5 | 5 | 3 | 5 | 5 | 4 | 5 | 5 | 5 | 5 | 5 | 5 | 5 | 4 | 5 | 3 | 4 | 5 | 5 | 5 | 3 | 5 | 5 | 4 | 5 | 5 | 5 | 5 | 3 | 5 | 5 | 5 | 3 | 5 | 5 | 5 | 3 | 5 | 3 | 5 | 4 | 4 | 3 |
| M | 3 | 5 | 4 | 5 | 5 | 3 | 4 | 3 | 4 | 4 | 5 | 3 | 5 | 5 | 4 | 5 | 3 | 3 | 3 | 3 | 4 | 4 | 3 | 3 | 3 | 4 | 4 | 5 | 5 | 4 | 4 | 5 | 4 | 5 | 3 | 3 | 3 | 5 | 5 | 2 | 5 | 3 | 3 | 3 | 4 | 4 |
| N | 4 | 4 | 4 | 4 | 5 | 5 | 4 | 4 | 4 | 4 | 4 | 4 | 4 | 4 | 4 | 4 | 4 | 4 | 4 | 5 | 4 | 4 | 4 | 4 | 4 | 4 | 4 | 4 | 4 | 4 | 4 | 4 | 4 | 5 | 5 | 5 | 5 | 5 | 5 | 5 | 5 | 5 | 5 | 5 | 4 | 4 |
| O | 3 | 3 | 3 | 3 | 5 | 3 | 3 | 3 | 3 | 3 | 3 | 3 | 3 | 3 | 3 | 5 | 3 | 3 | 3 | 5 | 4 | 3 | 3 | 3 | 3 | 3 | 5 | 3 | 3 | 3 | 3 | 3 | 3 | 3 | 3 | 3 | 3 | 5 | 5 | 3 | 5 | 5 | 3 | 3 | 3 | 3 |
