# Supplementary material for: Development of a Digital Health Literacy Assessment Framework for Older Adults: Delphi-Based Study
Source: J Med Internet Res. 2026 Apr 29;28:e82334. doi: 10.2196/82334 (PMC13127856; doi:10.2196/82334)
Supplement: Multimedia Appendix 2 [file jmir-v28-e82334-s002.docx]

Supplement materials: Survey Questionnaire on Digital Health Literacy Among Urban and Rural Residents in Sichuan Province

四川省城乡居民数字健康素养情况调查问卷

尊敬的女士/先生:

您好！非常感谢您在百忙之中填写这份问卷！我们是四川大学华西医院调查团队，本次调查的目的了解本市城乡居民的健康现状、疾病负担及电子健康素养等相关内容。我们向您承诺:本调查结果仅作研究之用，我们对您提供的资料绝对保密，本次调查对您没有任何不良影响，请您放心如实作答。感谢您的参与！

**第一部分:基本信息**

1.您的年龄是（ ）岁？ [填空题] *

_________________________________

2. 您的性别:（ ） [单选题] *

| A.男 |
| --- |
| B.女 |

3.您的居住地是：（ ） [填空题] *

_________________________________

4.您的婚姻情况:（ ） [单选题] *

| A.未婚 |
| --- |
| B.已婚 |
| C.离异 |
| D.丧偶 |

5. 请问您的民族:（ ） [单选题] *

| A.汉族 |
| --- |
| B.少数民族 _________________ *  若选“B”，请填写具体民族 |

6. 请问您的受教育程度:（ ） [单选题] *

| A.初中及以下 |
| --- |
| B.高中/职高/中专 |
| C.大专 |
| D.本科 |
| E.硕士及以上 |

7. 请问您从事的职业:（ ） [单选题] *

| A. 农民/农民工 |
| --- |
| B. 离退休人员 |
| C. 离退休人员 |
| D.其他 |

8. 您的基本医疗保险类型:（ ） [单选题] *

| A.职工基本医疗保险 |
| --- |
| B.城乡居民基本医疗保险 |
| C.未参保（请说明原因） _________________ * |

9.您家庭人口数:（ ） [单选题] *

| A.小于3人 |
| --- |
| B.3~5人 |
| C.5人以上 |

10.您的居住情况：（ ） [单选题] *

| A.与子女同住 |
| --- |
| B.与伴侣同住 |
| C.独居 |
| D.其他 _________________ *  若选“其他”，请注明具体情况 |

**第二部分:电子健康素养情况调查**

【概念解释】电子健康素养指的是个体基于电子资源平台，进行健康信息搜索、筛选、理解、评估及运用获得的健康信息解决相关健康问题的能力。

1.【个人信念】您对自我能力及互联网健康的信念和评价：（请选择跟您情况相符的选项）[矩阵单选题] *

| 条目 | 非常同意 | 同意 | 说不清 | 不同意 | 非常  不同意 |
| --- | --- | --- | --- | --- | --- |
| 我可以通过自己的努力来促进自身健康 | ○ | ○ | ○ | ○ | ○ |
| 互联网对于我获得健康资源的帮助很大 | ○ | ○ | ○ | ○ | ○ |
| 我觉得我可以利用网络来解答自己的健康问题 | ○ | ○ | ○ | ○ | ○ |
| 我对应用网络信息做出健康相关决定充满自信 | ○ | ○ | ○ | ○ | ○ |

2.【操作技能】以下操作对您来说难易程度如何？ [矩阵单选题] *

| 条目 | 非常  容易 | 比较  容易 | 一般 | 比较  困难 | 非常  困难 |
| --- | --- | --- | --- | --- | --- |
| 我能使用手机或电脑键盘输入文字或语音等内容 | ○ | ○ | ○ | ○ | ○ |
| 我能查找、关注健康类微信公众号或其他发布健康信息的新媒体平台 | ○ | ○ | ○ | ○ | ○ |
| 我会使用网络搜索健康相关信息 | ○ | ○ | ○ | ○ | ○ |
| 我能清楚地写出我的健康问题或担忧 | ○ | ○ | ○ | ○ | ○ |
| 我能清楚地表达我的症状和希望得到解决的健康问题 | ○ | ○ | ○ | ○ | ○ |
| 我可以通过多种网络渠道搜寻信息，来满足不同的健康信息需求 | ○ | ○ | ○ | ○ | ○ |
| 我能使用正确的词条/关键词查找我所需要的信息 | ○ | ○ | ○ | ○ | ○ |

3.【评判技能】以下操作对您来说难易程度如何？ [矩阵单选题] *

| 条目 | 非常  容易 | 比较  容易 | 一般 | 比较  困难 | 非常  困难 |
| --- | --- | --- | --- | --- | --- |
| 我能判断所找到的信息是否适合我的健康状况 | ○ | ○ | ○ | ○ | ○ |
| 我能从找到的所有信息中判断出相应的健康行为 | ○ | ○ | ○ | ○ | ○ |
| 我能确定我找到的信息是真实可靠的 | ○ | ○ | ○ | ○ | ○ |
| 我能确定信息是不是出于商业利益（如广告推销等） | ○ | ○ | ○ | ○ | ○ |
| 我能对比不同网站来判断信息的真实性 | ○ | ○ | ○ | ○ | ○ |
| 我会跟家人、朋友或医学专业人士分享与讨论检索到的健康信息以判断其是否可信 | ○ | ○ | ○ | ○ | ○ |

4.【应用技能】以下操作对您来说难易程度如何？ [矩阵单选题] *

| 条目 | 非常  容易 | 比较  容易 | 一般 | 比较  困难 | 非常  困难 |
| --- | --- | --- | --- | --- | --- |
| 我会在日常生活中应用的检索到的健康信息 | ○ | ○ |  | ○ | ○ |
| 我能将找到的信息用于做出与健康相关的决定（如营养、药物治疗、应急处理等） | ○ | ○ |  | ○ | ○ |

5.【安全与隐私】以下操作对您来说难易程度如何？ [矩阵单选题] *

| 条目 | 非常  容易 | 比较  容易 | 一般 | 比较  困难 | 非常  困难 |
| --- | --- | --- | --- | --- | --- |
| 我能判断和识别出危险和不安全的网站/社交软件 | ○ | ○ | ○ | ○ | ○ |
| 我能有效地保护自己或他人（家人/朋友等）的私人信息（如姓名、地址等） | ○ | ○ |  | ○ | ○ |
| 我能及时地清理网络浏览痕迹 | ○ | ○ | ○ | ○ | ○ |

6.【外部支持】您对外部支持情况的感知和评价:（请选择跟您情况相符的选项）

[矩阵单选题] *

| 条目 | 非常  同意 | 同意 | 说不清 | 不同意 | 非常  不同意 |
| --- | --- | --- | --- | --- | --- |
| 我能够利用当地的医疗卫生服务来满足我的健康需求 | ○ | ○ | ○ | ○ | ○ |
| 我能够利用社区组织的老年人智能技术教育培训来提高个人能力 | ○ | ○ | ○ | ○ | ○ |
| 我家或附近的养老服务机构或老年活动中心有宽带网络 | ○ | ○ | ○ | ○ | ○ |
| 我使用的手机/电脑等电子产品有专门便于老年人使用的功能（如大字体、大图标、大声音、远程输入等功能） | ○ | ○ | ○ | ○ | ○ |
| 我经常通过线上（如电话、微信等网络平台等）或线下（面对面）与家人互动交流 | ○ | ○ | ○ | ○ | ○ |
| 我经常通过线上（如电话、微信等网络平台）或线下（面对面）与亲戚/朋友/社区志愿者互动交流 | ○ | ○ | ○ | ○ | ○ |

7. 电子健康素养问卷(eHEALS)（请选择跟您情况相符的选项）[矩阵量表题] *

| 条目 | 非常  同意 | | 同意 | 说不清 | | 不同意 | | 非常不同意 | |
| --- | --- | --- | --- | --- | --- | --- | --- | --- | --- |
| 我知道如何上网查找有用的卫生资源信息 | ○ | | ○ | ○ | | ○ | | ○ | |
| 我知道如何利用网络来解答自己的健康问题 | ○ | | ○ | ○ | | ○ | | ○ | |
| 我知道网络上可以获取哪些卫生资源信息 | ○ | | ○ | ○ | | ○ | | ○ | |
| 我知道网络上哪里可以获取有用的卫生资源信息 | ○ | | ○ | ○ | | ○ | | ○ | |
| 我知道如何利用获取的网络卫生资源信息帮助自己 | ○ | | ○ | ○ | | ○ | | ○ | |
| 我具备评价网络卫生资源信息好坏的技能 | ○ | ○ | | | ○ | | ○ | | ○ |
| 我能够区分网络上卫生资源信息的质量 | ○ | ○ | | | ○ | | ○ | | ○ |
| 我对应用网络信息作出健康相关决定充满自信 | ○ | ○ | | | ○ | | ○ | | ○ |
